# Secretion rates and protein composition of extracellular vesicles released by cancer-associated fibroblasts after radiation

**DOI:** 10.1093/jrr/rrab018

**Published:** 2021-04-24

**Authors:** Rodrigo Berzaghi, Ashraful Islam, Turid Hellevik, Inigo Martinez-Zubiaurre

**Affiliations:** Department of Clinical Medicine, Faculty of Health Sciences, UiT-The Arctic University of Norway, 9037, Tromsø, Norway; Department of Clinical Medicine, Faculty of Health Sciences, UiT-The Arctic University of Norway, 9037, Tromsø, Norway; Department of Radiation Oncology, University Hospital of Northern Norway, Tromsø, Norway; Department of Clinical Medicine, Faculty of Health Sciences, UiT-The Arctic University of Norway, 9037, Tromsø, Norway

**Keywords:** cancer-associated fibroblasts (CAFs), exosomes, non-small cell lung carcinoma (NSCLC), ionizing radiation (IR), radiotherapy, extracellular vesicles (EVs), immunoregulation

## Abstract

Reciprocal communication between the malignant and non-malignant cellular elements in tumors is essential for cancer sustainability and plays an important role in the response of cancers to treatments. Some of this cellular crosstalk takes place via secretion of vesicles that are actively released into the extracellular space by most cell types in tumors. Recent studies have demonstrated radiation-induced changes in the secretion rate and composition of extracellular vesicles (EVs), with impact on radiation-related cellular communication. However, little is known about the effects of different radiation regimens on the release of EVs by cells of the tumor microenvironment. In this study, we provide a comprehensive molecular characterization of EVs released by cultured primary lung tumor fibroblasts. We explore the quantitative and morphological changes triggered by ionizing radiation (IR), delivered as a single dose of 18 Gy or three consecutive daily medium-doses of 6 Gy. Cancer-associated fibroblasts (CAFs) secrete EVs with sizes ranging from 80 to 200 nm, expressing some of the classical exosome markers. Exposing CAFs to a single-high radiation dose (1 × 18 Gy) or fractionated medium-dose did not alter the release of CAF-EVs. The protein composition of CAF-EVs was analyzed by LC-MS/MS proteomics and revealed that CAF-EVs are enriched with heat shock proteins, integrins, tetraspanins, proteinases, collagens, growth factors and an array of molecules involved in the regulation of cell migration and the immune system. Quantitative proteomic analyses revealed minor changes in the protein composition of CAF-EVs after radiation exposure. Taken together, this study presents original data on lung tumor CAF-EV composition and reveals that release and protein cargo of CAF-EVs are largely unaltered after exposing CAFs to IR.

## INTRODUCTION

As a cancer develops, an accompanying host stromal response evolves within and around the growing tumor. Already at very early stages of tumorigenesis, dynamic and reciprocal communication between malignant and host tissue cells is established and persist during all stages of cancer progression [1–3]. Cellular communication in the context of tumors may take place via direct cell–cell interactions, secretion of soluble molecules and/or the release of membrane-contained vesicles transporting bioactive molecules from donor cells to recipient cells. The so-called extracellular vesicles (EVs) comprise a heterogeneous group of particles composed of a lipid bilayer carrying transmembrane and cytosolic proteins, nuclear proteins, growth factors, lipids and nucleic acids including fragments of DNA and different RNA species [4–6]. EV is a generic term given to all categories of EVs that may vary in size (30–2000 nm in diameter) and intracellular origin, which depending on their biogenesis, are most frequently referred to as exosomes or microvesicles [5].

Among the host stromal constituents associated with tumors, cancer-associated fibroblasts (CAFs) are highly abundant and play important roles in the regulation of cancer progression [7, 8]. CAF is a broad name given to a complex and dynamically heterogeneous population of cells of mesenchymal lineage associated with tumors. They are found in the vicinity or direct contact with neoplastic cells and are frequently the dominant cell type within a solid tumor mass. CAFs have been shown to directly modulate the behavior of tumor cells or impact cancer progression indirectly by regulating angiogenesis and tumor immunity [9]. Additionally, CAFs are mainly responsible for ECM remodeling and desmoplastic reactions [10]. Although not fully demonstrated, it is believed that the different CAF subsets may exert different functions, in some cases tumor-promoting and in other cases tumor-restraining [7, 11, 12].

The EV-mediated crosstalk between CAFs and tumor cells has been studied in the past [13]. Exosomes and microvesicles released from CAFs have been reported to become internalized by cancer cells and contribute to cancer progression and metastasis [14, 15]. Earlier studies in different cancer models have demonstrated that CAF-EV cargo proteins and/or RNAs have the capacity to increase cancer cell migration and invasion [16, 17], as well as fuelling metabolic reprogramming [18]. Likewise, other studies have reported cancer cell therapy-resistance induced by CAF-derived exosomes [19–22] . Conversely, cancer cell-derived EVs may promote fibroblast activation and the induction of a myofibroblast phenotype, thus helping CAFs to acquire pro-tumorigenic properties [23, 24]. Furthermore, it has been proposed that tumor EVs could possibly interact not only with adjacent/neighboring fibroblasts but also with distant mesenchymal stromal cells (MSCs), thus contributing to distant MSC transformation and the setting of a prometastatic niche [18, 25].

Some studies have reported that ionizing radiation (IR) alters EV release in a dose- and time-dependent manner [26]. It has been suggested that this phenomenon occurs due to activation of stress-inducible pathways related to exosome secretion [27, 28]. Additionally, IR may provoke changes in EV compositions since the content of an EV primarily depends on type and state of a donor cell. However, original data regarding radiation-induced changes in EV composition is scarce and refers mainly to their proteome [28, 29]. Besides possibly affecting EV secretion, IR may also influence EV uptake by exposed recipient cells [30]. Collectively, most of the existing literature on this topic refers to IR effects on cancer cells, *in vitro* culture conditions, and radiation given in a single, relatively low dose (2–4 Gy) manner. Almost no information exists on the effect of IR on exosome-release by cells of the tumor microenvironment, and no studies compare the effects of different radiation regimens, i.e. high-dose vs low-dose IR. In this study, we present novel data on the composition of lung tumor-derived CAF-EVs and the influence of IR, given in different regimens, on lung CAF-EV secretion and associated protein cargo.

## MATERIAL AND METHODS

### Human material, CAF isolation, cell line and cultures

Human lung CAFs were isolated from freshly resected non-small cell lung carcinoma (NSCLC) tumor tissue from patients undergoing surgery at the University Hospital of Northern Norway (UNN), Tromsø. The Regional Ethical Committee of Northern Norway has approved the use of human material included in this study (REK Nord 2014/401; 2016/714; 2016/2307) and all patients provided informed written consent. The study includes lung tumor specimens from four different patients (Table 1). Experimentally, NSCLC-derived CAFs were isolated by enzymatic digestion of minced tumor tissues, and the outgrowth method. Culture expanded CAFs were characterized by the presence of lineage-specific markers, as described previously by our group [31]. Isolated CAFs were cultivated in Dulbecco’s modified Eagle’s medium (DMEM) (Sigma-Aldrich, St Louis, MO, USA) supplemented with 10% fetal bovine serum (FBS) and used for experimentation after the third and fourth passage (3- to 5-week-old cultures).

**Table 1:** Donor features corresponding to the CAF cell lines used in this study

Donors	Sex	Tumor type	T-size (mm)	T-stage
1	Male	Squamous cell carcinoma	35	pT2aN0Mx
2	Female	Invasive adenocarcinoma	21	pT1cN0Mx
3	Male	Squamous cell carcinoma	30	pT2aN0Mx
4	Male	Invasive adenocarcinoma	45	pT2bN2Mx

### Irradiation of cells and preparation of CAF-conditioned medium (CAF-CM)

Prior to irradiation, CAF cultures established in T-75 flasks were gently washed (4×) with phosphate-buffered saline (PBS) (pre-warmed at 37°C) and incubated in 10 mL serum-free medium (DMEM high glucose with ITS-Media Supplement). Adherent CAF cultures were irradiated with high-energy (MV) photons when 70–90% confluent, using a clinical Varian linear accelerator and two different radiation regimens; a single-high dose (1 × 18 Gy) or a fractionated regime (3 × 6 Gy) at 24 h intervals. Standard parameters for dose delivery were depth 30 mm, beam quality 15 MV, dose rate of 4.6 Gy/min and field sizes of 20 × 20 cm, gantry at 180°, as previously described [31]. On day three upon the first radiation dose, serum-free medium in culture flasks was replaced and conditioned for the next 48 h. After the incubation period, supernatants were collected, spun down by centrifugation (2000 × g, 4°C, 10 min) and then filtrated (Ø = 0.22 μm) for elimination of contaminating cell debris.

### Isolation of CAF-derived EVs

The CAF-conditioned medium was concentrated from 40 mL to a final volume of 5 mL using 100 kDa MWCO Vivaspin columns and centrifugation (3000 × g) (Sigma-Aldrich). The concentrated CAF-CM was then subjected to ultracentrifugation (110.000 × g, 4°C, 1.5 h). The resulting pellets were gently washed with PBS and ultracentrifuged once more (110.000 × g, 4°C, 1.5 h). CAF-EV-enriched fractions were resuspended in either: (i) PBS, for CAF-EVs characterization and quantification by flow cytometry, (ii) RIPA lysis buffer (CellSignal, Boston, MA, USA), plus Complete Protease Inhibitor Cocktail (Sigma-Aldrich), for quantification by Western blotting, and (iii) 1% sodium deoxycholate (SDS) in 100 mM triethylammonium bicarbonate (TEAB) buffer (ThermoFisher Scientific, Waltham, MA, USA), for mass spectrometry. If not used immediately, CAF-EVs were stored at −80°C (in PBS) upon isolation and before further analysis.

### CAF-EV characterization by immunogold labeling and transmission electron microscopy

CAF-EV samples were characterized by transmission electron microscopy (TEM) imaging of whole-mounted and immunolabeled EVs, essentially as described by Théry *et al.* [32]. Briefly, CAF-EV pellets were resuspended in (50–100 μL) 2% PFA. Next, 10 μL of fixed resuspended pellet was adsorbed onto Formvar-coated electron microscopy (EM) grids (20 min, 23°C), washed on PBS droplets, quenched for free aldehyde groups (PBS/50 mM glycine) and transferred to blocking buffer (PBS with 10% cold-water fish gelatin). Samples were then immunolabeled with primary antibody diluted (1:25) in a blocking buffer, then exposed to a bridging rabbit anti-mouse antibody and thereafter probed with a (5 nm) protein-A gold solution. Extensive washing on PBS/blocking solution was carried out between each step of the procedure. Finally, labeled grids were washed with ddH_2_O and then contrasted and embedded in a mixture (1:9) of (3%) uranyl acetate and (2%) methylcellulose on ice for 5 min.

Following this procedure, CAF-EVs were immunogold labeled with mouse monoclonal antibodies for exosome markers anti-CD81, anti-CD63 and anti-CD9 antibodies (# ab59477, ab59479, ab2215, Abcam, MA, USA), using rabbit anti-mouse IgG antibody (# M7023 Sigma Aldrich) as bridging antibody. Immunogold-labeled samples were imaged at high magnification with a JEOL JEM1010 transmission electron microscope (JEOL USA, Inc, Peabody, MA, USA) at 80 kV.

In addition, CAF-EVs were labeled for exosome markers by immunofluorescence and examined by high-resolution Super Illumination microscopy (SIM). To this end, CAF-EVs were adsorbed onto glass coverslips, then immunolabelled as before, using blocking solution consisting of (TBS with 3% BSA and 0.1% Tween-20) and antibody incubations (1 h, 23°C) in a humidity chamber. Upon extensive washing in blocking buffer, the primary antibody was probed with an Alexa Fluor 488-conjugated goat polyclonal anti-mouse secondary antibody (diluted in blocking buffer) (# ab150113, Abcam, MA, USA). Upon final washing with TBS-Tween-20 (0.1%), samples were visualized by a Zeiss LSM 800 microscope equipped with Airyscan module (Zeiss, Oberkochen, Germany). Extraction of single z-frame and maximum intensity projections were performed with ImageJ software.

### CAF-EV size distribution analysis

CAF-EV size distribution was determined by nanoparticle tracking analysis (NTA), using the NICOMP Submicron Particle Sizer Model 370 (NICOMP Particle Sizing system, CA, USA) according to the manufacturer’s protocol. Each measurement was done in triplicates (runtime 10 min; 23–24°C). Size distributions of EVs were expressed as mean diameter; the polydispersity index was determined using intensity-weighted distribution, whereas the zeta potential was determined on Malvern Zetasizer Nano, according to the manufacturer’s protocol (Malvern, Oxford, UK). The instrument was calibrated with Malvern Zeta potential transfer standard (−42 ± 4.2 mV), and measurements were performed at 25°C with an equilibration time of 180 sec. NTA was performed using NTA 3.2 software.

### Quantification of CAF-EVs by Western blotting

Whole-cell (CAF) extracts and CAF-EV lysates were prepared in RIPA buffer plus Complete Protease Inhibitor. Total CAF-EV lysate and 30 μg of whole-cell lysate were separated on 10% SDS-polyacrylamide gel electrophoresis (PAGE) and transferred onto a PVDF membrane. The membrane was blocked with 1% BSA in tris buffered saline, 0.1% Tween 20 (TBS-T) for 2 h at room temperature and then incubated (overnight, 4°C) with primary antibodies (anti-β-actin, # 8457, CellSignal; anti-CD81 and anti-CD63, Abcam) diluted 1:1000 (in TBS-T with 1% BSA). Subsequently, the membrane was washed 5× in TBS-T and then incubated with an anti-rabbit HRP-conjugated secondary antibody (diluted 1:250) for 1 h at room temperature. Finally, proteins transferred to the membrane were visualized with Enhanced Chemiluminescence at ImageQuant LAS 4000 CCD (GE Healthcare Bio-Sciences, PA, USA).

### Quantification of CAF-EVs by flow cytometry

CAF-EVs were quantified by flow cytometry on BD FACSAria III (BD Biosciences, San Jose, CA, USA). Briefly, flow cytometry light scattering and fluorescence performance (both sensitivity and resolution) were calibrated using a reference size fluorescent bead mix (diameters of 220 nm, 440 nm and 880 nm, # NFPPS-52-4 K Nano Fluorescent Size Standard Kit, Yellow, Flow Cytometry Grade, SpheroTech,). Cell-free tissue culture flasks treated identically as cell-containing flasks were used to identify potential impurities in samples. After flow cytometry calibration, the tubing was washed by 10% bleach followed by (0.22 μm pore size) filtering in PBS to remove any particles which might have adhered to the tubings. Tube-washing was performed at a sample flow rate of 15 μL/min for 120 s. Such alternating bleach/-PBS washes were included after every positive sample, to exclude carry-over of fluorescently positive events (EVs/fluorescent dye) between samples. The “clean” tubings and excluded noise signals were accessed by subsequent acquisition of filtered PBS sample at sample low-flow rate (2 μL/min), with 100-μL sample volume for 120 s. For vesicle quantification purposes, CAF-EVs-resuspended in 100 μL of PBS were labeled with anti-CD63 antibody (1:50, # 130-118-149. Miltenyi Biotec, Bergisch Gladbach, Germany) for 15 min at 4°C, whereupon labeled CAF-EVs were analyzed by flow cytometry, using a low flow-rate of 2 μL/min for 120 s (or until the data buffer was full up to 1,000,000 events). Collected data were analyzed using the FlowJo software, Ver.7.2.4 (Tree Star, Ashland, OR, USA).

### Quantitative and qualitative liquid chromatography-mass spectrometry (LC-MS/MS) analysis

Pellets with EVs were resuspended in 50 μL of 1% SDS in 100 mM TEAB buffer. Protein samples were then reduced in 5 mM dithiothreitol (Sigma-Aldrich) for 30 min at 70°C. Next, samples were alkylated by incubating with 375 mM iodoacetamide (ThermoFisher Scientific,) at 20°C for 30 min in the dark. Protein samples were collected as dry pellets after overnight precipitation in pre-chilled acetone at −20°C. Dry pellets were resuspended with 100 μL of 2 M urea (Sigma-Aldrich) in 50 mM TEAB buffer. Samples were next pre-digested for 6 h with 1:100 (w/w) LysC endopeptidase (# 125-05061, Wako Chemicals, Neuss, Germany) with 1 mM final concentration of CaCl_2_, followed by further dilution with 1 M urea (dissolved in 50 mM TEAB-buffer) and digestion overnight in 1:20 (w/w) trypsin (Promega). For protein/peptide precipitation, Trifluoroacetic acid (5 μL, 10%) (ThermoFisher Scientific) was added to each tube, followed by centrifugation of samples (16.000 × g, 10 min, 4°C). OMIX C18 tips were used for sample clean-up and concentration purposes. Samples containing 0.2% Formic acid (ThermoFisher Scientific) were loaded into a ThermoFisher Scientific EASY-nLC1000 system and EASY-Spray column (C18, 2 μm, 10 nm, 50 μm, 500 mm). Peptides were fractionated using a 2–100% acetonitrile (ThermoFisher Scientific) gradient in 0.1% Formic acid at a flow rate of 250 nL/min over 180 min. Separated peptides were analyzed using a ThermoFisher Scientific Q-Exactive mass spectrometer, and data were collected by Top10 method in data-dependent mode. The raw data were processed using MaxQuant (v 1.5.6.0) for label-free quantification (LFQ). MS/MS data were searched against the UniProt human database from November 2016 to yield protein identification [false discovery rate (FDR) = 0.01]. Parameters used for the search were: fixed modification, carbamidomethylation of cysteines; variable modifications, oxidation of methionine and acetylation of protein N-terminal; ion mass tolerance, 4.5 ppm; fragment mass tolerance, 20 ppm; charge states, 2+, 3+ and 4+; maximum missed cleavages, 2; enzyme specificity, trypsin; minimum number of unique peptides, 2. For statistical analysis of the identified proteins, Perseus 1.5.6.0 software (Max Planck Institute of Biochemistry, Germany) was used. All contaminants were filtered out before log10-transformation of data for further analysis, and the log10-transformed intensities were normalized by subtracting the median. Finally, Volcano plots for each comparison were generated to identify differentially expressed proteins between four different CAF donors, using FDR <0.01 (Fig. 5B).

### Βeta-galactosidase assay

Isolated CAFs (at passage 3) were seeded in DMEM supplemented with 10% FBS at a density of 20.000 cells per well in six-well plates and left for attachment and spreading for 24 h before irradiation. Five days post-irradiation, cultures were washed and fixed for 5–7 min at 20°C with paraformaldehyde (2%) and stained for β-galactosidase (5-bromo-4chloro-3-indolyl-B-D-galactopyranoside). Staining was achieved following instructions from the manufacturer; “Senescence Cells Histochemical Staining Kit” (# CS0030, Sigma-Aldrich). Randomly selected fields were photographed at 1000× magnification, using an Idea SPOT digital camera.

### TUNEL assays

DNA fragmentation and apoptosis in irradiated CAFs were examined by a fluorimetric TUNEL assay. For that purpose, CAF cells were plated at a density of 1 × 10^6^ cells/10-cm dish, and left for adherence for 24 h before exposure to IR, as described before [31]. Irradiated CAFs were further incubated for 48 h and then subjected to the TUNEL assay, using the APO-BrdU TUNEL assay kit with Alexa-Fluor 488 anti-BrdU antibody (# A23210 Invitrogen, Carlsbad, CA, EUA). Upon fixation and permeabilization, percentage of TUNEL-positive cells was analyzed by flow cytometry, using a BD FACSAria III flow cytometer (BD Biosciences, San Jose, CA, USA) and FlowJo software, Ver.7.2.4 (Tree Star, Ashland, OR, USA).

### Statistical analysis

All statistical analyses were performed using IBM SPSS statistics version 25 (IBM, Chicago, IL, USA). A comparison of data between the three experimental groups was analyzed using the non-parametric Kruskal-Wallis test, and significance values were adjusted by Bonferroni correction for multiple comparisons. The level of significance was set at *P*<0.05.

## RESULTS

### Purification and characterization of EVs from CAF culture supernatants

**Fig. 1.**
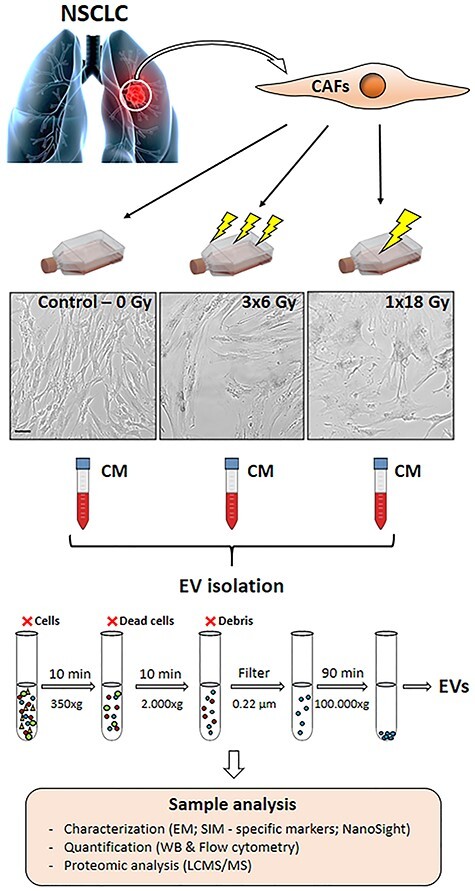
Schematic representation of the experimental workflow used in the study. CAFs were isolated from fresh NSCLC specimens and grown in exosome-free culture media. CAF cultures were submitted to single high-dose (1 × 18 Gy) or fractionated (3 × 6 Gy) radiation regimens. CAF-EVs were isolated by sequential centrifugations and ultracentrifugations of conditioned medium from irradiated and non-irradiated CAFs. Purified EVs were resuspended and characterized as follows: CAF-EVs morphology analyzed by immuno-gold labeling for EV-markers and imaged by TEM and SIM; protein expression of specific EV-markers quantified by Western blotting and total CAF-EVs was quantified by flow cytometry. The CAF-EV protein cargo was analyzed by LC-MS/MS. Scale Bar = 15 μm.

**Fig. 2.**
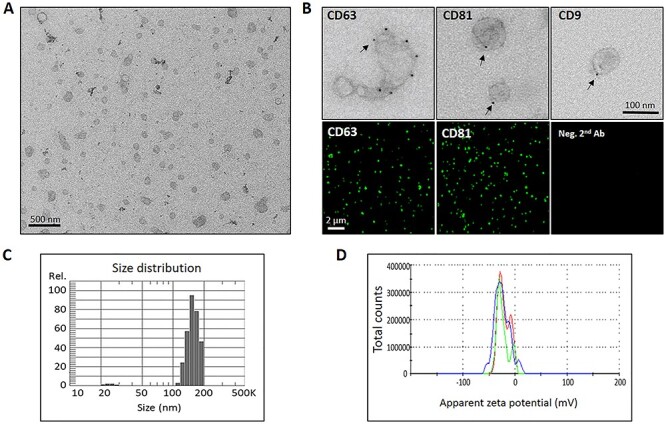
Characterization of EVs released by CAFs. (a) Representative (low magnification) EM micrograph of isolated CAF-EVs, (b) Immunogold and immunofluorescence microscopy analysis of EVs for specific exosomal markers CD9, CD63 and CD81, (c) Size distribution of the generated EVs particles, as determined by a Nano Particle Tracking assay, and (d) Zeta Potential Distribution Analyzer of released CAF-EVs. Scale bars = 200 nm (TEM); 100 nm (immunogold), and 2 μm (SIM).

CAF-EVs were isolated and purified from CAF-CM by sequential centrifugations, filtration and ultracentrifugation. Next, the isolated vesicles were characterized by their integrity and morphology, purity and size distribution, using qualitative immunogold labeling and TEM as well as size-determination by NanoSight (size) tracking analysis (Fig. 1). To evaluate integrity and morphology, CAF-EV samples were examined by TEM. These image analyses showed a homogeneous mixture of EVs with typical round- or cup-shaped bilayer structures with various size ranges, which was mostly appearing as isolated rather than aggregated particles (Fig. 2a). Isolated vesicles were positively labeled for anti-CD63, anti-CD81, and anti-CD9 antibodies by immuno-gold labeling. The surface distribution of EV-specific markers varied between the antibody markers. Immunofluorescent images showed that the majority of EVs express both CD63 and CD81 on the surface (Fig. 2b). CAF-EVs size distribution analysis revealed an EV population that was size-wise heterogeneous with diameters ranging from 80 to 200 nm (mean diameter of 156 nm, ± 18.2), and vesicles being negatively charged, with a zeta potential of −21 mV (± 6.4) (Fig. 2c and d). These data demonstrate that CAFs secrete a heterogenic population of vesicles that differ in size and expression levels of specific EV markers.

### Irradiated CAFs undergo premature cell senescence but not apoptotic cell death

**Fig. 3.**
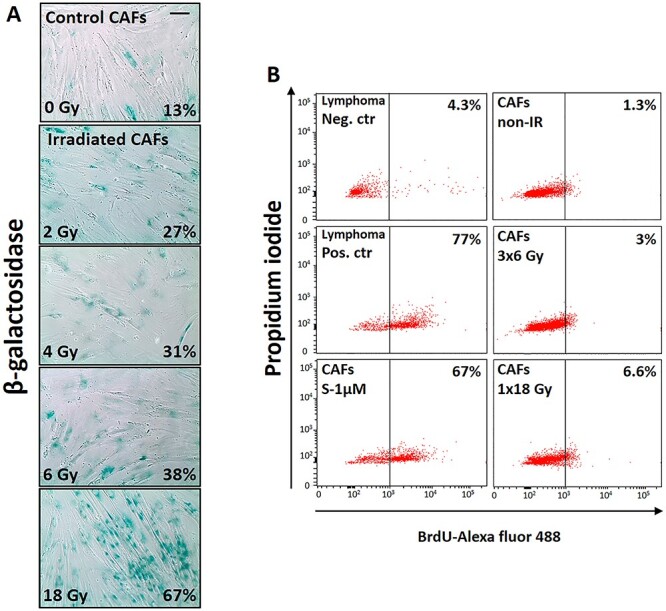
RT effects on cell senescence and apoptosis. Primary CAF cultures were checked for senescence (β-Galactosidase assay,—panel A) and apoptosis (TUNEL assay—panel B) at day five after first radiation exposure. High extent of premature cell senescence (blue-stained cells), but not apoptosis was induced by high-dose radiation regimen. Scale Bar = 15 μm.

The extent of IR-induced cellular senescence in CAFs has been reported previously by us [31, 33]. Induction of cell senescence was visualized in irradiated CAFs by analyzing for β-galactosidase activity, five days after single radiation doses of 2 Gy, 4 Gy, 6 Gy and 18 Gy. Irradiated cultures showed prominent changes in cell morphology and induction of β-galactosidase staining, suggesting that a large proportion of irradiated CAFs entered growth arrest by activating premature cellular senescence mechanisms. The senescence response was more pronounced after exposure to a single-high dose (1 × 18 Gy) than after low/medium doses (Fig. 3a). In addition, cell survival and apoptosis induction in irradiated CAFs was assessed by flow cytometry. As expected, only marginal apoptotic responses were observed in CAFs examined 48 hours post-IR exposure, and measured by the apoptosis-determining TUNEL assay **(**Fig. 3b). Comparison between positive controls, including human lymphoma cells treated with camptothecin—(77%, ± 9.1), or CAFs treated with pro-apoptotic agent staurosporine—(67%, ± 0.71), with either control CAFs (1.3%, ± 1.91), fractionated irradiated CAFs (3%, ± 3.04) or CAFs irradiated with a single high-dose (6.6%, ± 1.2) showed no significant enhancement of apoptotic rates in irradiated CAFs (Fig. 3b). Collectively, these results indicate that IR exposure to lung CAFs triggers cellular senescence in a dose-dependent manner, but in line with previous results, IR clearly does not induce apoptotic cell death in CAFs.

### IR does not alter CAF-EV secretion

**Fig. 4.**
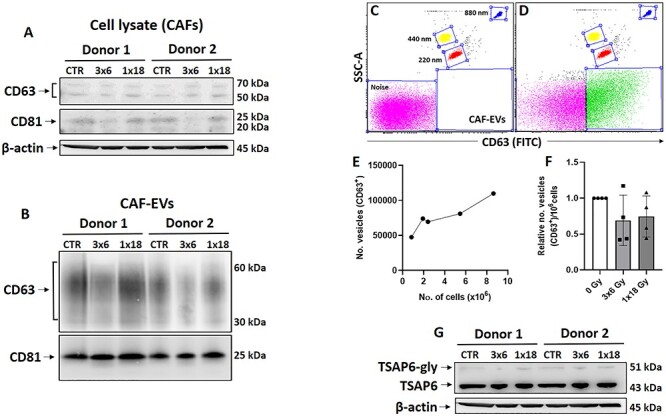
Quantification of EVs levels released by irradiated or non-irradiated CAFs. Western blot analysis, using anti-CD63 and anti-CD81 antibodies on whole-cell lysates (a) and total EV-lysates released by irradiated and non-irradiated CAFs (b). (c) Dot plot representation of filtered cell-free medium sample and reference size beads (blue dots, 880 nm; yellow dots, 440 nm; and red dots, 220 nm) used to define the gating strategy to select the EVs population based on size scattering and CD63 (FITC) positive staining. (d) Dot plot from a random CAF-EV sample showing EVs gated (green dots). Noise signal (pink dots) was excluded from EVs analysis. (e) Flow cytometry quantification showing the relation between the number of vesicles vs the number of CAF in dishes. (f) Quantitative analysis of EVs isolated from irradiated and non-irradiated CAFs by flow cytometry. Data represent mean (± SD) values from four different CAF donors. Results were normalized against cell number in cultures. (g) Quantitative Western blot analysis of TSAP6 expression in whole-cell lysates. Anti-(beta) actin was used as a control for equal loading of CAFs whole-cell lysate.

To investigate the influence of IR on the secretion of CAF-EVs, we used a double approach comprising of Western blots and flow cytometry. Amounts of specific EVs markers CD63 and CD81 on total CAF-EVs lysates and whole-cell lysates from irradiated and non-irradiated CAFs were analyzed by Western blot. As expected, the analysis showed low CD63 and CD81 protein levels on whole-cell lysates as compared to total CAF-EVs lysates from two different CAF donors (Fig. 4a and b). Importantly, no significant differences in expression of CD63 and CD81 were observed in EVs secreted by CAFs irradiated with high single-dose or fractionated medium-doses as compared to non-irradiated CAFs (Figure 4b). In addition, we quantified EVs using flow cytometry. Cell-free medium samples and reference size beads were used to define the gating strategy to quantify CAF-EVs (Fig. 4c). EVs were gated based on size scattering and CD63 (FITC) positive staining (Fig. 4d, green dots). To assess the accuracy of the methodology, we purified EVs from CAF cultures containing doubling number of cells and quantified the corresponding purified EV fractions by flow cytometry. This method revealed that the amount of EVs vs number of cells follows a close to linear progression (Fig. 4e). Moreover, the quantitative analysis from four different CAF donors showed no significant differences in the amount of EVs secreted by irradiated and control CAFs (Fig. 4f). Results were normalized against the number of cells in cultures. Last, no significant differences in the expression of TSAP6 (transmembrane protein tumor suppressor-activated pathway 6), a protein involved in stress-induced pathways of exosome secretion [27], were observed on CAF cells lysates from irradiated and non-irradiated CAFs (Fig. 4g). Collectively, these data indicate that IR applied in different regimens does not affect substantially the secretion of EVs from CAFs.

### IR does not alter the protein cargo in CAF-EVs

**Fig. 5.**
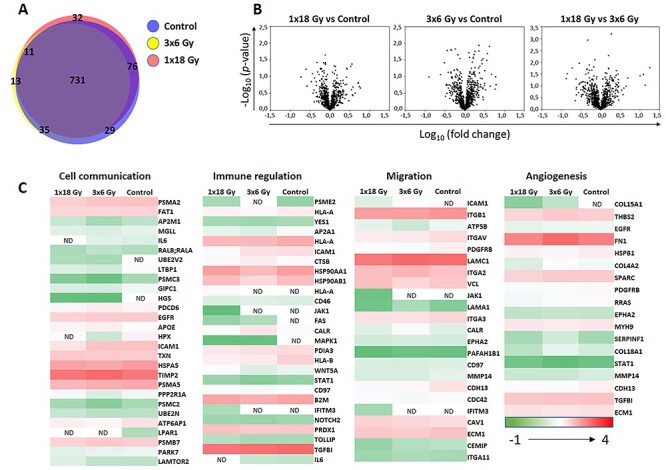
Comparative high-throughput protein expression analysis by LC-MS/MS. In (a), the Venn diagram depicts percentage of identified proteins shared among three experimental groups: EVs from untreated CAFs (blue circle), EVs from CAFs exposed to fractionated radiation (3 × 6 Gy) (yellow circle), or EVs from CAFs exposed to single high-dose radiation (1 × 18 Gy) (pink circle); In (b), Volcano plots illustrates the results of three sets of statistical comparisons made between groups. These plots show each protein with –Log10 (p-value) and Log10 of fold-change of the comparison on the Y-axis and X-axis, respectively. No significant differences in protein regulations were observed between groups. In panel (c), comparative heat maps showing identified proteins on EV samples involved in cell communication, immune regulation, migration and angiogenesis. Low-expressed proteins are indicated in green, whereas highly expressed proteins are in red.

A comparative analysis of the EV protein profile between irradiated and non-irradiated CAFs was analyzed by LC-MS/MS proteomics. Only proteins identified in EVs from at least three of four independent CAF donors were included in these analyses. Proteomic analysis identified a total of 871 proteins in EVs derived from non-irradiated CAFs; 790 proteins in EVs derived from fractionated irradiated CAFs (3 × 6 Gy); 850 proteins in EVs derived from single high-dose irradiated CAFs (1 × 18 Gy). The results are indicating that the large majority of identified proteins (n = 731, 83.9%) are overlapping between the three groups (Fig. 5a). Among the shared identified proteins in our data set, we find proteins belonging to specific pathways that regulate cancer development and progression, such as tetraspanins (CD151, CD63 and CD81), growth factor receptors (EGRF, PDGRF and TNF-R), cell surface receptors (CDR2, CD44, CD97, CD59, CD109), integrins (A1-A6, A11 and B1, B3, B5), growth factors (BMP1, HGF, IGF2, MIF, IL-6, ICSF-1, PDGF, TGF-β, CXCL12/SDF-1, VEGF), immune regulators (PD-L1, HLA-1, HSP70 and 90, CD276, CD58, Calreticulin, Protein S100, Galectins), cell migration regulators (TSP1, 2 and 3, VCAM, ICAM, VEGF, Angiopoietin) and extracellular matrix remodeling proteins (ADAMs, HSPG, TIMPs, Collagens, SERPHs, MMP1, 2, 14 and 19). The complete lists of proteins that were only detected on EVs secreted by either non-irradiated or irradiated-CAF (3 × 6 Gy or 1 × 18 Gy) are shown in Supplementary Table 1. Interestingly, the list of proteins exclusively found on EVs from high-dose irradiated CAFs contains the GTPase Rab31, a Rab-5 subfamily member involved in the trafficking of early endosomes [34] and presumably exosome release (review in [35]). In qualitative terms, the analyses also reveal that the antigen-presenting molecule MHC-I is expressed on EVs from non-irradiated and high-dose irradiated CAFs, but not in the fractionated IR group. Quantitative analysis of protein expression was performed using the LFQ approach (Fig. 5b). Three Volcano plots where generated, representing all possible comparisons between experimental groups. The Volcano plots show differentially expressed proteins by plotting Log10 of the fold-change on the X-axis, and –Log10 of the *P*-value on the Y-axis, for each group-comparison. These results showed no significant differences in protein expression between the three experimental groups. Protein profiles belonging to specific pathways, e.g. cell communication, immune regulation, migration and angiogenesis, were also compared among irradiated and non-irradiated CAF-derived EVs, but no significant differences in protein expression were observed among the three groups (Fig. 5c). These results indicate that IR exposure to CAFs does not modify substantially the protein cargo of CAF-EVs.

## DISCUSSION

An increasing number of studies highlight the contribution of CAF-EVs in paracrine cellular communication processes in different types of cancers [15, 36–38]. However, little is known about the secretion and functions of stroma-derived EVs in the context of radiotherapy. In the present study, we perform a comprehensive characterization of protein content of lung tumor CAF-EVs and investigate the effects of IR (applied in different regimens) on EV secretion and associated protein cargo. The outcomes have revealed that: (i) CAFs secrete a heterogeneous population of EVs expressing classical exosomal markers; and (ii) CAF-EVs carry a myriad of factors that could have an impact on tumor aggressiveness and tumor immunoregulation. Importantly, we have demonstrated that neither high-dose radiation nor repeated medium-dose (6Gy)-fractionated radiation induces substantial changes in EV secretion rates or EV protein content.

It has been demonstrated in different cancer models that CAF-EVs play an important role in the crosstalk between CAFs and cancer cells, thereby contributing to carcinogenesis and tumor development [15–17, 37]. Like other cell types in tumors, CAFs secrete exosomes (small vesicles with diameter in the range 40-150 nm) and microvesicles (MVs—generally larger; 100–1000 nm) [37, 39]. In most instances, the protein content of CAF-EVs is comparable between different tumor types and includes proteins normally enriched in exosomes such as CD9, CD63 and CD81 and proteins involved in exosome biogenesis like ESCRT-associated proteins, Alix, TSG101, HSC70 and HSP90β. However, the effects exerted by CAF-EVs on tumor cells may differ depending on EV-specific membrane proteins. In breast cancer, for example, CD81-positive exosomes derived from CAFs promotes breast cancer cell motility and metastasis [40]. On the other hand, CAFs from scirrhous-type gastric cancer secretes exosomes CD81-negative/CD9-positive, which can promote cancer cell migration and invasion by activating the MMP2 signaling pathway [38]. In our study, the morphology and size distribution analysis revealed that CAF-EVs was composed probably by a mixed population of exosomes and microvesicles, highly enriched in tetraspanins CD81 and CD63, but not much of CD9. In line with our findings, others have demonstrated that about 50% of vesicles secreted from human dendrtic cells (DCs) are rather large (Ø = 100-200 nm) [41].

Taking into account that the presence of apoptotic bodies could interfere with our results, we found that there are marked discrepancies in the literature about the characterization of apoptotic bodies, making it difficult to accurately distinguish them from exosomes and microvesicles [42]. In the case of irradiated (and non-irradiated) lung tumor CAFs, we believe that the isolated pull of EVs do not contain apoptotic bodies since the extent of CAF-apoptosis is very low even in cells irradiated with high radiation doses (6.6% vs 1.3% in non-irradiated cells).

External stimuli and stress conditions, including IR, may evoke changes in EV secretion and composition [28, 29]. Abramowicz *et al*. [43] showed that exposure of UM-SCC6 cells (HNSCC tumor cells) to different radiation doses (2, 4 and 8 Gy) affects the pattern of exosome-specific markers CD63 or CD81, whereas others remained unchanged (e.g. CD9 or TSG101). In analyses by proteomics, the authors identified over 1200 exosomal proteins, including 425 up-regulated by IR and 47 down-regulated. Among overrepresented pathways associated with IR-affected proteins were those involved in the DNA damage response and repair of double-strand breaks. An analysis of EVs released by BHY and FaDu cells irradiated with 6 Gy dose revealed 39 up-regulated and 36 down-regulated proteins and an overrepresentation of processes associated with regulation of cell motility when compared with EVs released from non-irradiated cells [44]. In our study with human lung CAFs, we have not observed significant differences in the protein composition of EVs after exposure to high radiation doses. Lehmann *et al.* [28] demonstrated that IR-induced cell cycle arrest (upon 4 Gy) was associated with a senescence-associated increase in exosome release. Previous reports have suggested a role of p53 activation in the regulation of exosome release after DNA damage [27]. The authors highlight the participation of TSAP6, a p53-regulated gene product, in the enhancement of exosome production in cells undergoing p53 response to stress. We have shown previously that high-dose IR induces an intensive degree of senescence in CAFs [31]. However, in this study, we demonstrate that IR did not modulate CAF-EV release. Our results also suggest that the extent of radiation-induced DNA damage in CAFs is not sufficient to activate expression of TSAP6. Regarding EV cargo, previous reports have demonstrated radiation-induced changes in protein and mRNA composition of EVs released by cancer cells [26, 43]. These previous studies have been performed on tumor cells and operating with single, relatively low radiation doses. In our study, using radiation doses corresponding to hypofractionated regimens often delivered to NSCLC tumors in the clinics, we have not found significant changes in the protein content of CAF-EVs, despite the clear induction of cellular senescence. The minor group of differentially expressed proteins in qualitative analyses correlates with endoplasmic reticulum stress responses induced by IR, and proteins involved in intracellular trafficking (Supplementary Table 1).

**Fig. 6.**
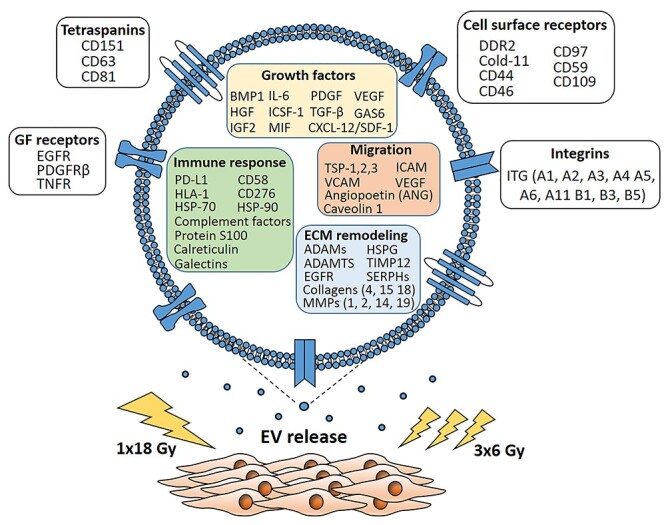
Schematic representation of the main findings of the study. CAFs exposed to a single-high radiation dose or fractionated medium-dose did not change the levels of EV in supernatants, as compared to untreated CAFs. Proteomic analyses of CAF-EV cargo uncovered the presence of relevant molecules that could be involved in the regulation of tumor growth, invasion and tumor immunomodulation. By comparative quantitative proteomics, we show that IR does not alter substantially the protein cargo of secreted CAF-EVs.

Furthermore, our high-throughput proteomic approach reveals comparable expression of proteins belonging to specific pathways that regulate cancer development and also anti-tumor immune responses, e.g., PD-L1, MHC-I, TGF-β, HLA-1, HSP70 and −90, CD276, CD58 and calreticulin in EVs from both irradiated and control CAFs. In the three experimental groups, we find comparable amounts of identified proteins (871 in control group, 790 in 1× 18 Gy group and 850 in 3 × 6 Gy group), from which 85% of proteins are overlapping in the three groups. Previous studies using proteomics to analyze CAF-Evs composition have underscored the important role of specific EV-associated proteins in regulating tumor cell behavior. Thus, Uchihara *et al.* [45] found that annexin A6 in CAF-EVs plays a pivotal role in network formation and drug resistance of gastric cancer cells in the ECM via activation of β1 integrin-focal adhesion kinase (FAK)-YAP. In an oral tongue squamous cell carcinoma model, Principe *et al*. [46] highlight the role of exosomal MFAP5, a protein component of extracellular microfibrils, in cancer cells growth and migratory capacity, whereas in a pancreatic cancer model, Leca *et al*. [14] demonstrate the role of annexin A6, LDL receptor-related protein 1 (LRP1) and thrombospondin 1 (TSP1) from CAF-EVs in improved cancer cell survival and migration. In our LC/MS–MS analyses, we were able to identify annexin A6, MFAP5 and LRP1 in NSCLC CAF-EVs, however, expression of these proteins was unchanged after IR.

Direct effects of CAF-EVs on tumorigenic properties of cancer cells and in cancer therapeutic resistance have been reported by others. Recent studies have conclusively demonstrated that tumor-derived EVs induce anti-tumor immunosuppression through PD-L1 [47–49]. In esophageal cancer, for example, CAF-derived exosomes containing several microRNA species play a role in tumor progression, influencing tumor cell adhesion, endocytosis and cell–cell junctions [50]. You *et al.* [39] demonstrated that exosomes derived from Snail-1-transfected CAFs significantly enhance EMT processes on lung cancer cells. Additionally, Sun *et al.* [51] demonstrated that CAF-derived exosomes transport miR-382-5p to oral squamous cell carcinoma (OSCC) cells and contribute to increased OSCC cell migration and invasion. We have previously shown that conditioned medium from lung tumor CAFs and irradiated CAFs exert powerful immunosuppressive effects on peripheral blood lymphocytes and monocyte-derived macrophages [33, 52], and those supernatants from irradiated CAFs attenuate the migratory capacity of endothelial cells [53]. In parallel experiments performed with A549 lung tumor cells and peripheral blood lymphocytes, we could not see effects of CAF-EVs on the proliferative or migratory capacity of cells (data not shown), indicating that, at least in these models, the inhibitory effects exerted by CAF-CM are rather originated from soluble signal molecules and not from EVs. Collectively, the proteomic analyses of CAF-EV samples in this study uncover the presence of multiple factors that could directly contribute to tumor growth regulation or tumor immunoregulation (summarized in Fig. 6), but their actual regulatory capacity still needs to be demonstrated in functional assays and by models *in vivo*.

## CONCLUSION

The present study provides novel and relevant information on lung tumor fibroblast-derived EVs, and aid for a better understanding of the complex interactions within the tumor stroma and the dynamic evolution of these interactions during tumor progression and in response to radiotherapy. Our study has focused on the characterization of the proteome of lung CAF-EVs, however EV-associated nucleic acids including mRNA and non-coding RNAs such as miRs and lncRNAs could have an impact on the transcriptome of recipient cells and should be considered in future studies. Finally, our study demonstrates that IR does not exert changes in the release of EVs from CAFs and does not have an impact on their protein cargo. Future experimentation on the functional effects of CAF-EVs using tumor cell lines and *in vivo* models would be needed to clarify the role of lung tumor CAF-EVs in tumor regulation, immunoevasion and radiation responses.

## Supplementary Material

Supplementary_table_1_rrab018

## Data Availability

Source data and reagents are available from the corresponding author upon reasonable request.
